# Economics methods in Cochrane systematic reviews of health promotion and public health related interventions

**DOI:** 10.1186/1471-2288-6-55

**Published:** 2006-11-15

**Authors:** Ian Shemilt, Miranda Mugford, Michael Drummond, Eric Eisenstein, Jacqueline Mallender, David McDaid, Luke Vale, Damian Walker, The Campbell & Cochrane Economics Methods Group (CCEMG)

**Affiliations:** 1 School of Medicine, Health Policy & Practice, University of East Anglia, Norwich, UK; 2 Centre for Health Economics, University of York, UK; 3 Duke University Medical Center, North Carolina, USA; 4 Matrix Research & Consultancy Ltd, London, UK; 5 LSE Health and Social Care, London School of Economics, UK; 6 Health Economics Research Unit, University of Aberdeen, UK; 7 John Hopkins Bloomberg School of Public Health, Baltimore, USA

## Abstract

**Background:**

Provision of evidence on costs alongside evidence on the effects of interventions can enhance the relevance of systematic reviews to decision-making. However, patterns of use of economics methods alongside systematic review remain unclear. Reviews of evidence on the effects of interventions are published by both the Cochrane and Campbell Collaborations. Although it is not a requirement that Cochrane or Campbell Reviews should consider economic aspects of interventions, many do. This study aims to explore and describe approaches to incorporating economics methods in a selection of Cochrane systematic reviews in the area of health promotion and public health, to help inform development of methodological guidance on economics for reviewers.

**Methods:**

The *Cochrane Database of Systematic Reviews *was searched using a search strategy for potential economic evaluation studies. We included current Cochrane reviews and review protocols retrieved using the search that are also identified as relevant to health promotion or public health topics. A reviewer extracted data which describe the economics components of included reviews. Extracted data were summarised in tables and analysed qualitatively.

**Results:**

Twenty-one completed Cochrane reviews and seven review protocols met inclusion criteria. None incorporate formal economic evaluation methods. Ten completed reviews explicitly aim to incorporate economics studies and data. There is a lack of transparent reporting of methods underpinning the incorporation of economics studies and data. Some reviews are likely to exclude useful economics studies and data due to a failure to incorporate search strategies tailored to the retrieval of such data or use of key specialist databases, and application of inclusion criteria designed for effectiveness studies.

**Conclusion:**

There is a need for consistency and transparency in the reporting and conduct of the economics components of Cochrane reviews, as well as regular dialogue between Cochrane reviewers and economists to develop increased capacity for economic analyses alongside such reviews. Use of applicable economics methods in Cochrane reviews can help provide the international context within which economics data can be interpreted and assessed as a preliminary to full economic evaluation.

## Background

Evidence on 'what works' is central to the formulation of public sector policy and practice. High quality systematic reviews can provide robust and comparatively inexpensive evidence of what is known about intervention effectiveness, which may often be more likely to convince decision-makers than evidence from single studies [1,2].

The Cochrane Collaboration and Campbell Collaboration are parallel international organisations committed to summarising and providing access to evidence on the effects and other aspects of interventions using systematic review methods. This involves the systematic identification, critical review and synthesis (typically using a meta-analysis) of evidence on main clinical effectiveness (incorporating adverse events and complications) – evidence primarily extracted from randomised controlled trials. The aim is to maximise the precision of estimates of effectiveness (or, alternatively, to minimise bias in such estimates) by selecting studies based on predetermined criteria, including study quality. Cochrane focuses on health care interventions, whilst Campbell focuses on education, criminal justice and social welfare interventions. There is nevertheless some overlap in focus of interest between the Collaborations where interventions, client groups, important outcomes, or aspects of review methodology cross these policy areas.

Cochrane and Campbell reviews are not currently required to incorporate an economics component. However, it is increasingly accepted that provision of evidence on intervention costs *alongside *evidence on effectiveness can significantly enhance the relevance and usefulness of Cochrane and other systematic reviews as a component of the basis for decision-making by managers and policy makers [3]. Evidence regarding the economics of interventions is useful at various stages of knowledge about their effectiveness. The relationship between expected changes in effects and costs determines the type and degree of added value from the intervention. Clearly if outcomes are improved or not changed and costs are reduced, a technology will be economically efficient. However, where there is likely to be a high overall cost of improving outcomes, decision makers will need to consider if this is the best use of resources. Finally, where outcomes are actually worse as a result of a form of care, the cost of changing practice to avoid that form of care also needs to be considered. Cochrane and Campbell reviewers increasingly encounter economics studies conducted alongside the randomised controlled trials and other studies included in their reviews. These factors have led to increased numbers of Cochrane reviews, and a small number of Campbell Reviews, incorporating some economics aspects.

Economic evaluation is the comparative analysis of alternative courses of action in terms of both their costs (resource use) and consequences (effectiveness) [4]. We use the term 'evidence-based economic evaluation' to distinguish forms of full economic evaluation based on systematic review and research synthesis methods from those based on a single primary study, such as economic evaluations conducted alongside randomised controlled trials. Although the latter can clearly be of high research quality, they may not include all available and relevant evidence in their analyses for policy decisions. Evidence-based economic evaluation is increasingly used to inform policy decisions falling under the auspices of organisations with national responsibilities for health technology assessment and guidance on the prevention and treatment of ill health (e.g. National Institute for Health and Clinical Excellence (NICE); US Center for Disease Control and Prevention (CDC); Canadian Agency for Drugs and Technology in Health (CADTH, formerly CCHOTA); Australian Pharmaceutical Benefits Advisory Committee (PBAC)).

Whilst the details of methodological requirements for such studies vary across these agencies [5], evidence-based economic evaluation essentially sets out to estimate, or model, the joint distribution of costs and effects resulting from a health care intervention or programme, compared to its alternatives, within a defined population, by systematically identifying and synthesising the best available sources of data on five key parameters: main clinical effectiveness, adverse events and complications; baseline clinical events; resource use and unit costs; and utilities (or other measures of preferences for health states) [6]. The aim is to maximise the precision of estimates of these parameters (or, alternatively, to minimise bias in their estimation), so that residual uncertainty associated with estimates of incremental cost-effectiveness (joint costs and effects) is minimised. A useful decision-aid, in the form of graphical presentation of costs and effectiveness outcomes, is a 'cost-effectiveness plane' (or 'cost-effectiveness matrix') [7].

A well-conducted meta-analysis of randomised controlled trials, with direct comparison between alternative interventions, has been proposed as the least-biased source of evidence to inform ranges of values for the main clinical effectiveness parameter in an evidence-based economic evaluation [6,8]. Indeed, for this reason, companion systematic reviews of effectiveness studies (or 'effectiveness reviews') often provide a natural vehicle for evidence-based economic evaluations, such as those undertaken for NICE. Similarly, Cochrane (or Campbell) reviews incorporating a meta-analysis can provide an unbiased source for the effectiveness parameter in an economic evaluation (for a full economic evaluation, the effectiveness review will need to be supplemented by additional searches to inform ranges of values for the other key parameters in the economic model).

As such, the interfaces between evidence-based economic evaluation and Cochrane and Campbell review methods are bilateral and multifaceted. Whilst on one hand a Cochrane or Campbell review can provide useful and applicable data for an evidence-based economic evaluation, full economic evaluation studies and other economics studies (e.g. costing studies) can also be included in a critical review of the extant economics literature that may be undertaken as an integrated component of a Cochrane (or Campbell) review. Some methodological research has already been undertaken to examine the use of Cochrane and other effectiveness reviews and the Cochrane Library in evidence-based economic evaluations [6,9]. However, patterns of use of economics methods within Cochrane reviews remain unclear.

The Campbell & Cochrane Economics Methods Group (CCEMG) was formally approved and registered as a methods group for The Cochrane Collaboration in 1998, and as a joint methods group with The Campbell Collaboration since 2004. CCEMG's aims and scope, reported elsewhere, encompass development of evidence-based economic evaluation methods and economics advice for reviewers [10].

This paper reports a rapid review of existing Cochrane reviews which focus on the effectiveness of health promotion and public health interventions. The main aim of the study, undertaken on behalf of CCEMG, was to examine approaches to incorporating economics studies and data that have been utilised in such reviews to date. A better understanding of these issues can help to inform the development of evidence-based guidance for reviewers on methods for making reviews more useful to economic decisions.

The reason for choosing to look at Cochrane reviews was because, being a much newer collaboration, fewer Campbell reviews are yet published. The topic area 'health promotion and public health' was chosen because many health promotion and public health interventions, whilst aiming to influence health outcomes and resources, are organised across sectors (e.g. health, education, social care, criminal justice) and are likely to have important impacts on outcomes and resource use beyond the health sector, and because a key aim of the study was to pilot a review methodology in preparation for a systematic review of economics methods in both Cochrane Collaboration and Campbell Collaboration systematic reviews, encompassing reviews of complex and multi-sector interventions, as well as interventions in health care, health promotion and public health, mental health, education, criminal justice and social welfare.

## Methods

The *Cochrane Database of Systematic Reviews*, *Cochrane Library *(2005, Issue 3) was searched using a search strategy for economic studies. This was adapted from an established search strategy used by the UK Centre for Reviews and Dissemination to identify potential economic evaluations within Ovid CD-ROM versions of Current Contents – Clinical Medicine, Medline and CINAHL databases as part of the process of maintaining the NHS Economic Evaluation Database [11], published as part of the Cochrane Library. The strategy was used to search the title, abstract and keyword fields within records of both completed reviews and review protocols held in the Cochrane Database of Systematic Reviews [12]. The search was conducted on 4^th ^August 2005. The full search strategy was as follows:

(econom*, cost*, pric*, pharmacoecon*, cost NEXT (effectiveness, utili*, benefit, minimi*, energy, oxygen, metabolic), (expenditure NOT energy), (value NEAR/2 money), budget*, preference, qaly, (quality NEXT adjusted), utility, utilities, financ* NEXT (management, support, organized))

The Cochrane Health Promotion and Public Health Field maintains an online list of Cochrane reviews and review protocols identified as relevant to health promotion and public health [13]. Reviews and review protocols were included if they appeared on both the latter list and the list of record titles retrieved by the search. Full reports of included reviews and review protocols were assessed to establish details of the economics component of each review. Data describing the economics component of reviews were extracted, coded, tabulated, analysed qualitatively and summarised by one researcher, working independently.

For those reviews which aim to incorporate economics studies, initial focussed searches for additional, relevant full economic evaluation studies (not included in the review) were conducted in the NHS Economic Evaluation Database, using search strategies that were developed by combining search terms used in the corresponding review with MeSH terms used to index the corresponding review.

## Results

**Figure 1:**
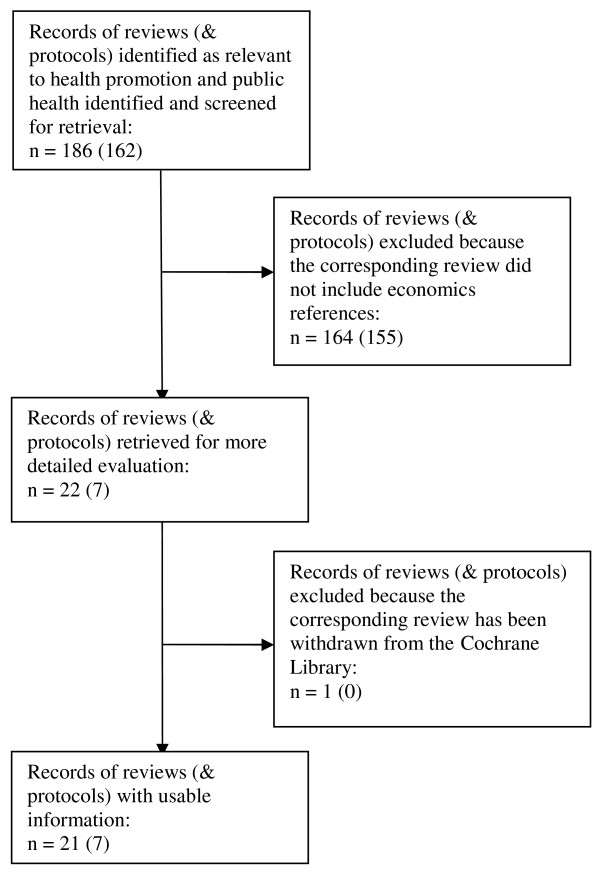
Quorum statement flow diagram.

A 'Quorum Statement' flow-diagram is included as Figure 1. The initial search of the *Cochrane Database of Systematic Reviews*, to identify economic references in existing reviews and protocols, identified 483 records out of 4041 records held in the database. One record was excluded at this stage since the corresponding review has been withdrawn. Four hundred and five records contained current, completed Cochrane reviews. Seventy-seven records contained protocols for Cochrane reviews – reviews which are registered but not yet completed.

Twenty-one of the 405 completed reviews retrieved by the initial search are also listed as relevant to health promotion and public health [[22-42] in Table 1]. As such, approximately 11% of the total number of completed reviews identified as relevant to health promotion and public health contain economics references (21 of 186). These 21 reviews spanned a wide range of health promotion and public health topics (e.g. mental and social health; population screening; prevention of infectious disease; prevention of injuries; sexual health; cardiovascular health). Additionally, 7 of the 77 review protocols retrieved by the initial search are also listed as relevant to health promotion and public health [[43-49] in Table 1]. This represents approximately 4% of the total number of review protocols identified as relevant to health promotion and public health (7 of 162).

**Table 1:** Results

**No.**	**Approach to incorporating economics studies and/or data**				**Analysis of economics studies and/or data**			**Other issues relevant to economics methods**				
	Review aims to include economics studies	Review aims to incorporate formal economic evaluation methods	Review includes a specific economic question	Review specifies one or more economic variable(s) as a review outcome	Review identified no primary studies incorporating economic evaluation methods or other economic analyses	Review includes a narrative summary of economics data extracted from included studies	Review incorporates a quantitative synthesis of economic variables extracted from included studies	Review incorporates a search of one or more specialist databases of economics studies (e.g. NHS EED)	(Further) potentially relevant economic evaluation studies identified by initial NHS EED searches for potential inclusion in an updated review	Reviewers suggest that economic analyses of the intervention were not appropriate due to a lack of conclusive evidence on intervention effectiveness	Overall effectiveness results (primary outcomes)	Reviewers highlight a need for primary economic evaluation studies

22	•		•		•						No evidence	•
23	•		•			•			•	•	No clear evidence of effectiveness	
24									n/a		Evidence of a positive effect	
25	•			•		•					Evidence of a positive effect	•
26					•				n/a	•	No clear evidence of effectiveness	
27	•		•	•	•						No clear evidence of effectiveness	•
28									n/a		Evidence of a positive effect	
29									n/a		No evidence	•
30	•			•	•				•		Good evidence of effectiveness	•
31						•					Evidence of mixed effectiveness	•
32											No evidence	•
33	•			•		•		•			Evidence of mixed effectiveness	•
34									n/a		Evidence of mixed effectiveness	•
35	•			•		•			•		No evidence	
36									n/a		Some evidence of ineffectiveness	
37									n/a		Weak evidence of a positive effect	
38									n/a		Strong evidence of effectiveness	•
39	•			•	•						No evidence	
40	•			•	•			Not Clear	•	•	No evidence	
41												
42	•		•		•		•			•		

**Total**	**10**	**0**	**4**	**7**	**6**	**6**	**0**	**1**	**5**	**3**	-	**11**

43					n/a	n/a	n/a	n/a	n/a	n/a	n/a	n/a
44					n/a	n/a	n/a	n/a	n/a	n/a	n/a	n/a
45					n/a	n/a	n/a	n/a	n/a	n/a	n/a	n/a
46	•		•		n/a	n/a	n/a	n/a	n/a	n/a	n/a	n/a
47	•		•	•	n/a	n/a	n/a	n/a	n/a	n/a	n/a	n/a
48	•		•	•	n/a	n/a	n/a	n/a	n/a	n/a	n/a	n/a
49	•		•	•	n/a	n/a	n/a	n/a	n/a	n/a	n/a	n/a

**Total**	**4**	**0**	**4**	**3**	-	-	-	-	-	-	-	-

### Approaches to incorporating economics studies or data

The principal findings from this rapid review are summarised in Table 1. It is evident that none of these 21 completed reviews, or 7 review protocols, incorporates formal economic evaluation methods (i.e. a cost-consequences, cost-effectiveness, cost-utility, or cost benefit analysis). That is, none of the reviews includes an aim to model the joint distribution of incremental costs and effects resulting from the interventions studied, compared to relevant alternatives, based on a synthesis of the results of studies included in the review.

Ten of 21 completed reviews do aim explicitly to incorporate economics studies and/or data into the review (4 of 7 protocols include this explicit aim). However, none of these ten reviews describe either a specific methodological approach (e.g. a systematic review of economic evaluation studies) or specific methods (e.g. search strategies, critical appraisal, inclusion criteria, analytic methods) underpinning incorporation of such studies and data.

One review protocol does include a clear description of the specific methodological approach that will underpin incorporation of economics studies and data. Ilic *et al *include, as a secondary objective, to "conduct a systematic review of studies regarding the economic evaluation of screening for prostate cancer. This will aim to identify the cost effectiveness, cost utility and cost-benefit of mass screening for prostatic cancer" [48].

It is, however, possible to establish some details of the approaches pursued in the ten completed reviews that explicitly aim to incorporate economics studies and data. Four of these ten reviews include a specific economic question or objective to be addressed in the systematic review (alongside the main focus on intervention effectiveness) [22,23,27,42]. For example, Hey and Perrera [23] ask, "What are the cost implications of providing incentives and competitions for smoking cessation?", whilst Ashworth *et al *[27] ask, "Which exercise programmes are the most cost-effective?" Note that the economic question formulated by Hey and Perrera [23] implies a concern with evidence related to the distribution of costs associated with the interventions being studied, whilst the question formulated by Ashworth *et al *[27] implies a concern with evidence related the joint distribution of costs and effects associated with the interventions, or cost-effectiveness (as did the objective in the Ilic *et al *protocol [48]).

Seven of the ten reviews aiming to incorporate economics studies and data [25,27,30,33,35,39,40] include one or more economic variables – measures of cost, resource use or service utilisation – as outcomes of interest in the review, alongside other 'effectiveness' outcomes. For example, Kujan *et al *[39] include "costs" in a set of secondary outcomes alongside "incidence of oral cancer or potentially premalignant oral lesions", "mortality at three or more years" and "stage of diagnosis" ("oral cancer specific mortality" is selected as the primary outcome of interest in this review). Similarly, Chilvers *et al *[40] include "capital expenditure", "total cost of care per tenant or respondent" and "total health costs per tenant or respondent" in a set of secondary 'economic outcomes'. It is of note that in this second example [40], specific components of intervention 'costs' are clearly described, whilst in the first [39], it is comparatively unclear which elements of 'costs' will be considered. All economic variables specified as outcomes in these Cochrane reviews imply a concern with evidence related to the distribution of costs (or resource use) associated with the interventions being studied, as opposed to evidence on cost-effectiveness.

None of the ten completed reviews aiming to incorporate economics studies and data appears to include use of supplementary search strategies designed specifically to capture economics studies and data other than those conducted alongside those studies captured in searches specified for the effectiveness review. Also, only one of these ten reviews explicitly includes a search of one or more specialist electronic databases of economics studies [33] (one protocol, incorporating plans for a systematic review of relevant economic evaluation studies, signals an intention to include such a search [48]). The extent to which search strategies for relevant economics studies and data extended beyond those studies identified and retrieved for the effectiveness review is therefore unclear.

### Analysis of economics studies and data

Five of the 10 completed reviews aiming to incorporate economics studies and data (plus one other review which did not explicitly seek such studies or data [26]) indicate that no such relevant studies or data are identified [22,26,27,30,39,40]. This would clearly preclude any form of analysis intending to utilise such studies or data. However, some reviews – including at least one review which sought, but did not identify any such studies or data [30] – are likely to have missed potentially relevant economics studies that could have been identified through searches using the NHS Economic Evaluation Database (NHS EED) [14] and/or other specialist electronic databases of economics studies.

For example, our own searches of NHS EED, conducted to inform this rapid review, identified a small number of additional potentially relevant economic evaluation studies that had not been identified in the corresponding Cochrane review. One or more additional, potentially relevant economic evaluation studies were identified for 5 of the 10 reviews that include an aim to incorporate economics studies and data [23,30,35,40,42] (although in two of these five cases, a single additional economic evaluation has been published since the last substantive update of the corresponding Cochrane review [23,40]). These additional economics evaluation studies were based either on a review and synthesis of primary studies, or on effectiveness data from sources other than the types of studies (primarily randomised trials) targeted for the Cochrane effectiveness review. It is also worth highlighting that a majority (although not all) relevant economic evaluation studies which had been identified across these 21 Cochrane reviews have a corresponding full abstract record in the NHS Economic Evaluation Database.

The other 5 completed reviews aiming to incorporate economics studies and data (with the addition of one other review which did not explicitly seek such studies or data [31]) include a narrative summary of relevant findings in economics studies identified in the review [23,25,31,33,35,42]. For example, Forbes *et al *[33] include a summary of cost data reported alongside two included randomised controlled trials – one appears to be a costing study which presents data on total estimated costs of the interventions for each intervention group; another appears to be an economic evaluation which presents data on the estimated incremental costs of the interventions per additional diagnostic test (cervical smear test) performed, compared with usual care. Lees *et al *[35] include a narrative summary of the general conclusions of three economic evaluations of neonatal screening programmes, whilst Gillespie *et al *[31] include a narrative summary of cost-effectiveness estimates (cost per fall prevented) extracted from five included randomised controlled trials that had incorporated economic evaluations.

None of the reviews incorporating a narrative summary which focuses on the costs of the interventions (e.g. presenting data on the incremental costs of resource inputs required for an intervention) includes adjustment of such data for currency and inflation, and only one presents data in such a way that would allow such adjustments to be undertaken subsequently [31]. Also, none of these narrative summaries include critical appraisal of the methodological quality of included economics studies, or commentary on the economic questions, costing methods and analytic viewpoints adopted in such studies. None of the completed reviews incorporates a quantitative synthesis of measures of cost or resource use, extracted from included primary studies (e.g. meta-analysis).

### Other issues relevant to economics methods

Three completed reviews draw attention to the point that economic analyses of the interventions are not currently appropriate, due to inconclusive or methodologically weak evidence, or a lack of evidence of intervention effectiveness (in terms of primary review outcomes) [23,26,40]. More generally, only 6 of the 21 Cochrane reviews considered in this rapid review find that the intervention(s) studied show evidence of 'effectiveness', or 'a positive effect' (including those which show 'good evidence', or 'strong evidence' of effectiveness) [24-26,30,38,42]. Three of these 6 reviews aim to include economics studies or data [25,30,42], of which one [42] has been found to have missed a small number of potentially relevant economic evaluation studies. The other 15 reviews either conclude that there is: no evidence [22,29,32,35,39,40]; no clear evidence of effectiveness [23,26,27]; weak evidence of effectiveness [37,41]; evidence of mixed effectiveness (i.e. the intervention was found to be effective in some groups but not necessarily in others) [31,33,34]; or some evidence of ineffectiveness [36].

Eleven of 21 completed reviews highlight a need for future primary research incorporating economic evaluation methods in order to establish evidence on intervention cost-effectiveness [22,25,27,29-34,38,42]. This included two reviews for which additional, potentially relevant economic evaluations have been identified through initial searches of the NHS economic evaluation database (conducted to inform this rapid review) [30,42].

Finally, three other reviews amongst the 21 considered in this rapid review were retrieved by the initial search of the Cochrane Library because the review covers discussion of one or more relevant economic aspects of the populations or interventions being studied [24,28,37]. In most cases the scope of this discussion relates to economic factors as determinants of epidemiology, patterns of intervention provision or uptake, or potential economic consequences of interventions. For example, Shepherd *et al *highlight the higher incidence of cervical cancer amongst women of lower socio-economic class than amongst middle class women in Britain [28], whilst Zoritch *et al *highlight that different levels of day-care provision in part reflect cultural and economic interests concerning the welfare of children and the need to promote mothers' participation in paid work [24].

## Discussion

This rapid review has shown that relatively few Cochrane systematic reviews relevant to health promotion and public health incorporate an economics dimension. It is also clear however that a sizable minority of Cochrane reviewers are aware that their topic of review has economic implications and wish to report this aspect. The review has further shown that where such reviews do incorporate economics studies and data, methodological approaches vary considerably, and the methods used are not consistently reported. The extent to which these principal findings are reflected across all Cochrane reviews may usefully be explored further.

It was not anticipated that the Cochrane reviews included in this study would widely incorporate formal economic evaluation methods. The Cochrane Collaboration has always aimed to produce high quality reviews of effects of health care interventions in order to support decisions which also need information from other sources. However, since Cochrane reviews aim to have international applicability, it is not currently clear how incorporation of full economic evaluation methods could be constructively achieved within such reviews [15]. This is because full economic evaluation involves estimation of the joint distribution of incremental costs and effects (cost-effectiveness) accruing from an intervention, compared to relevant alternatives, for specific populations, jurisdictions, or settings. Such estimates are sensitive to variability across settings in local prices (cost), the organisation of care (resource use) and values attached to specific health related outcomes (e.g. utilities) [16-18] and this may limit the generalisability or transferability of such estimates across settings (e.g. across different nations). Conversely, there is a general belief that relative clinical effectiveness (or 'relative risk reduction') is not influenced by jurisdiction and setting (although absolute risk reduction is dependent on baseline levels of risk in a given population, which is variable across settings). Whilst this naturally militates against the incorporation of formal economic evaluation methods into Cochrane reviews, it does not preclude the use of data from Cochrane reviews in parallel, or subsequent jurisdiction specific economic evaluation studies. It should also be noted that for the same reasons, there are currently no agreed upon methods for combining incremental cost-effectiveness, cost-utility or cost-benefit ratios from multiple studies.

The results also indicate that this set of Cochrane reviews rarely select studies on the basis that they meet criteria for being classified as full economic evaluations, such as those defined by Drummond *et al *[4,19]. Rather, studies are selected based on inclusion criteria set for the effectiveness review (with a focus on randomised trials). As such, this set of Cochrane reviews considers only those economics studies conducted alongside studies eligible for inclusion in the effectiveness review. This may exclude relevant data contained in full economic evaluations based on reviews of existing trials, those based on effectiveness data from sources other than randomised controlled trials, and other economics studies, or partial economic evaluations (e.g. costing studies).

Use of searches specifically tailored to the retrieval of relevant economics studies, and searches of specialist databases, such as NHS EED, would help to address this situation. It has been shown in this study that incorporation of such searches can prove fruitful. Additionally, reviewers considering the economic perspective should consider undertaking classification of the economics studies encountered in a review, and critical appraisal of their methodological quality, using a purpose-specific, established checklist prior to the final decision to include or exclude such studies [19,20].

Although the CCEMG has existed for nearly a decade, evidence-based guidance on handling economic issues in Cochrane Reviews has not been covered specifically in the Cochrane Reviewers' Handbook. Reviewers have had access to workshops at annual meetings and to informal advice from the members of the economics methods group. Perhaps linked to this lack of agreed approach, the methods used to derive cost and resource use, or cost-effectiveness estimates in primary studies are not consistently critiqued in this set of Cochrane reviews.

Some of the reviews report economic outcomes that were measured within one or more trials. Economics studies will vary in their results because the economic question and analytic viewpoint varies between studies, and so the variables included and the values given to them are specific to the study [15]. Formal critique of included economics studies is required to clarify, for the users of reviews, whether such estimates consist of comparable, or homogeneous, cost, or resource use components.

Even where it is not appropriate to combine cost or resource use data, cost or resource use estimates from several primary studies can provide a range of values for cost and resource use parameters in a sensitivity analysis that may be conducted as part of a parallel or subsequent economic evaluation based on the review. Allied to this, estimates of monetary cost could be adjusted for currency and inflation, in order to facilitate comparison of estimates across several included studies.

More generally, the intended scope of overall analytic approaches to economics studies and data is often unclear in this set of Cochrane reviews. Although we found none in this study, other Cochrane reviews include measures of cost and/or resource use as review outcomes with the intention of extracting these data from relevant economics studies and producing pooled estimates of incremental costs or incremental resource use using meta-analysis [e.g. [21]], including a test for heterogeneity (this is the same basic approach as that taken to the meta-analysis of effect-size and adverse effects data in Cochrane and other effectiveness reviews). When presented alongside the pooled estimates of effect-size (i.e. separate estimation of the distribution of costs and the distribution of effects), these data can provide an indication of the quadrant of the 'cost-effectiveness plane' that an intervention is likely to fall within. This is potentially useful information that can help determine whether further economic evaluation is warranted.

However, it is not clear in this set of Cochrane reviews how those including economic variables originally intended to analyse them. It is therefore not possible to judge whether intended aims were fulfilled with respect to the economics components of the reviews. This situation could be improved with more consistent reporting of intended methods.

Finally, it could be argued that economic decisions should only be made on the basis of strong evidence of intervention effectiveness and that, by implication, Cochrane reviews should concentrate on establishing evidence of effectiveness before any decision to include an economics dimension. However, at the point of planning a Cochrane review, it is likely that economic decisions are being, or have already been taken about the technology being studied. It therefore seems to the authors that there is likely to be additional value to end-users in a systematic review which refers to the existing economics evidence, whatever the current status of the effectiveness evidence. This may allow decision makers using the review to place the technology on the cost-effectiveness plane [7]. For example, if a review concludes that there is evidence of no statistically significant difference between comparator interventions (clinical effectiveness), then critical review of existing economics evidence can help to establish, given similar clinical effectiveness, which intervention is less costly?

## Conclusion

Cochrane reviews of the effects of health care interventions are used in economic decisions in a wide range of settings, and many review groups are aware of this. Within the Cochrane Collaboration, diverse methods for addressing economics issues have evolved, and we are convinced, from the results of this review, of the value of developing guidance for reviewers wishing to incorporate economics aspects in their review.

Evident variability across approaches to economics studies and data in Cochrane reviews, all within a single, broad topic area, indicates a clear need for evidence-based guidance for Cochrane reviewers on whether and how to incorporate economics methods into the systematic review process, at different levels. Such guidance will need to promote a consistent approach to the reporting of the economics components of such reviews. There is a also need for increased dialogue between Cochrane reviewers and economists to develop the capacity for economic analyses alongside Cochrane reviews, for example to decide on the need to consider economic aspects, to set their economic findings within a discussion of the economic issues around a technology, and to refer to other studies of the economics of the technology being evaluated.

The current study has also indicated that Cochrane reviews may require a specific approach to guidance on methods, distinct from generally available advice on economic evaluation and Health Technology Assessment, given the aim of the Cochrane Collaboration to provide an international source of evidence on effects of health related interventions. However, whilst economics methods in a global context. are likely to be distinct from those underpinning evidence-based full economic evaluations, they nevertheless feature on the same methodological continuum.

That is, the analytic framework provided by economic evaluation can help Cochrane reviewers to formulate an integrated systematic review of relevant economics studies, alongside the review of effectiveness studies, but which need not necessarily be restricted to the economics components of included effectiveness studies. This type of full critical review, including coverage of all the economic analyses of the economics of interventions, can provide users of the review with the international context within which economics data can be interpreted and assessed as a preliminary to use of evidence-based economic evaluation methods.

## Competing interests

The author(s) declare that they have no competing interests.

## Authors' contributions

IS conceived of and designed the study, conducted the study, interpreted the findings and drafted the manuscript. MM conceived of the study, participated in its design and helped draft the manuscript. MD, EE, JM, DM, LV and DW conceived of the study and helped draft the manuscript. MM, MD, EE, JM, DM, LV and DW are co-convenors of The Campbell & Cochrane Economics Methods Group (CCEMG), a joint Methods Group for The Cochrane Collaboration and The Campbell Collaboration. CCEMG is currently developing methodological research and evidence-based guidance for systematic reviewers on how to incorporate economics evidence into the systematic review process, which have helped to inform interpretation of this study. All authors have read and approved the final manuscript.

## Pre-publication history

The pre-publication history for this paper can be accessed here:


